# Natural Killer Cells-Produced IFN-γ Improves Bone Marrow-Derived Hepatocytes Regeneration in Murine Liver Failure Model

**DOI:** 10.1038/srep13687

**Published:** 2015-09-08

**Authors:** Lu Li, Zhutian Zeng, Ziping Qi, Xin Wang, Xiang Gao, Haiming Wei, Rui Sun, Zhigang Tian

**Affiliations:** 1 Hefei National Laboratory for Physical Sciences at Microscale and School of Life Sciences, University of Science and Technology of China, Hefei, Anhui 230027, China; 2 Collaborative Innovation Center for Diagnosis and Treatment of Infectious Diseases, State Key Laboratory for Diagnosis and Treatment of Infectious Diseases, First Affiliated Hospital, College of Medicine, Zhejiang University, Hangzhou, Zhejiang 310003, China; 3 The Key Laboratory of National Education Ministry for Mammalian Reproductive Biology and Biotechnology, Inner Mongolia University, Hohhot 010070, China; 4 Model Animal Research Center, Nanjing University, Nanjing 210061, China

## Abstract

Bone-marrow transplantation (BMT) can repopulate the liver through BM-derived hepatocyte (BMDH) generation, although the underlying mechanism remains unclear. Using fumarylacetoacetate hydrolase–deficient (*Fah*^*−/−*^) mice as a liver-failure model, we confirmed that BMDHs were generated by fusion of BM-derived CD11b^+^F4/80^+^myelomonocytes with resident *Fah*^*−/−*^ hepatocytes. Hepatic NK cells became activated during BMDH generation and were the major IFN-γ producers. Indeed, both NK cells and IFN-γ were required for BMDH generation since WT, but not NK-, IFN-γ–, or IFN-γR1–deficient BM transplantation successfully generated BMDHs and rescued survival in *Fah*^*−/−*^ hosts. BM-derived myelomonocytes were determined to be the IFN-γ–responding cells. The IFN-γ–IFN-γR interaction contributed to the myelomonocyte–hepatocyte fusion process, as most of the CD11b^+^ BMDHs in mixed BM chimeric *Fah*^*−/−*^ hosts transplanted with a 1:1 ratio of CD45.1^+^ WT and CD45.2^+^ *Ifngr1*^*−/−*^ BM cells were of CD45.1^+^ WT origin. Confirming these findings *in vitro*, IFN-γ dose-dependently promoted the fusion of GFP^+^ myelomonocytes with *Fah*^*−/−*^ hepatocytes due to a direct effect on myelomonocytes; similar results were observed using activated NK cells. In conclusion, BMDH generation requires NK cells to facilitate myelomonocyte–hepatocyte fusion in an IFN-γ–dependent manner, providing new insights for treating severe liver failure.

Liver failure, characterized by massive hepatocyte death and severely impaired liver function, is a major life-threatening condition in patients with hepatitis-, drug-, or metabolic disorder–induced liver injury^1^,^2^. Orthotopic liver transplantation (OLT) remains the most efficient approach for treating liver failure, but this approach is largely limited by the supply of donor livers^3^. Many studies have provided evidence demonstrating that transplanted donor bone marrow cells (BMCs) give rise to multiple types of liver epithelial cells after transplantation, including hepatocytes, oval cells, and duct epithelium^4^,^5^,^6^, indicating that BM may possess a great clinical potential for treating severe liver failure.

BM-derived hepatocytes (BMDHs) were first discovered in the livers of patients that received cross-gender donor BM transplantation (BMT), raising the possibility that donor BMCs could generate hepatocytes to repopulate damaged liver^5^. Further investigation into this phenomenon using mouse models determined that BMDH generation occurred through a cell-fusion mechanism between BMCs and resident hepatocytes rather than through direct differentiation from hematopoietic stem cells (HSCs)^7^,^8^. Within BMCs, the myelomonocyte subpopulation is recognized as the major fusion partner with hepatocytes during BMDH generation^9^,^10^, but the specific cell type within this subpopulation remains unknown along with the other factors that contribute to BMDH generation *in vivo*. Granulocyte colony-stimulating factor (G-CSF)–mediated mobilization of HSCs is also known to significantly promote BMDH generation^11^, but whether other immune components, like lymphocytes or cytokines, are involved in BMDH generation is not well understood.

Innate immune cells are predominant in the liver and respond rapidly during liver injury and regeneration^12^. As an important component of innate immune cells, NK cells represent 50% of the lymphocytes in the human liver and function as important regulators of liver regeneration. On one hand, hepatic NK cells have been shown to inhibit liver regeneration by secreting IFN-γ and activating STAT1 in hepatocytes^13^; on the other hand, they have also been shown to promote liver regeneration through extracellular ATP in partially hepatectomized mice^14^. Thus, although the precise role of NK cells in liver reconstitution is controversial, investigating whether and how they participate in BMDH-dependent liver repopulation is of great interest.

Several mouse models including carbon tetrachloride (CCl_4_) treatment, glucose-6-phosphatase catalytic subunit (G6PC) deficiency, or 2-acetylaminofluorene/partial hepatectomy have been used to study BMDHs^11^,^15^,^16^,^17^. However, autologous hepatocyte regeneration in these mouse models can interfere with studying the mechanisms underlying BMDH generation. A more convenient and appropriate tool to investigate the mechanisms underlying BMDH generation may be the fumarylacetoacetate hydrolase–deficient (*Fah*^*−/−*^) mouse model. These mice are ideal tools for this purpose because the lack of Fah results in metabolite accumulation that induces hepatocyte apoptosis, eventually leading to spontaneous liver failure if these mice are not supplemented with NTBC (2-[2-nitro-4-tifluoro-methylbenzyol]-1, 3-cyclohexanedione), an agent that blocks HPPDO upstream of Fah and prevents metabolite accumulation^18^. Theoretically, BMT with WT BM could rescue all resident *Fah*^*−/−*^ hepatocytes that would normally have died after NTBC withdrawal, allowing us to investigate the mechanisms underlying BMDH generation.

In this study, we found that NK cells are essential for BMDH generation in *Fah*^*−/−*^ mice. Moreover, this NK cell function is dependent on their production of IFN-γ, which directly promotes myelomonocyte–hepatocyte fusion. From these findings, we propose that manipulating NK cells or IFN-γ may be beneficial for effective BMT-based therapy of severe liver failure.

## Results

### NK cells are indispensable for BM-supported liver regeneration during hepatic failure

**Figure 1:**
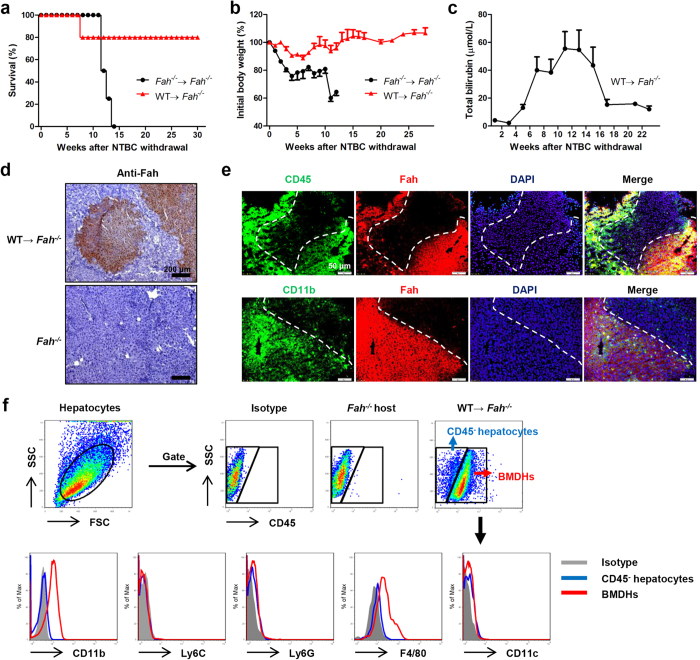
BMT rescues liver failure through the generation of hepatocytes in *Fah*^*−/−*^ mice. Survival rate (**a**) and body weight (**b**) of *Fah*^*−/−*^ mice transplanted with *Fah*^*−/−*^ (*n* = 4) or WT (*n* = 5) BMCs. (**c**) Total bilirubin levels in sera of *Fah*^*−/−*^ mice transplanted with WT BMCs after NTBC withdrawal. (**d,e**) Liver tissues of WT BM-transplanted *Fah*^*−/−*^ mice were collected 20 weeks after NTBC withdrawal and stained for Fah by immunohistochemistry (**d**, scale bar, 200 μm) and immunofluorescence (**e**, scale bar, 50 μm). The boundary of Fah^+^ hepatocyte area is indicated by dashed white line. (**f**) FACS analysis of BMDHs and *Fah*^*−/−*^ hepatocytes 27 weeks after NTBC withdrawal. Gray shadows represent staining by the isotype control. Data are expressed as the mean ± SEM.

In order to begin exploring the mechanism underlying how BMDHs develop and function to repopulate the liver after BMT to treat hepatic failure, *Fah*^*−/−*^ mice were transplanted with WT BMCs and monitored for more than 30 weeks after NTBC withdrawal. WT, but not *Fah*^*−/−*^, BMCs promoted survival (Fig. 1a), rescued severe weight loss (Fig. 1b), and decreased the elevated levels of total bilirubin (Fig. 1c) in *Fah*^*−/−*^ mice, indicating that hepatocytes were directly and efficiently re-established in these mice after transplantation of WT BMCs. Indeed, robust formation of BM-derived, Fah^+^ hepatocytes was consistently observed in the livers of transplanted *Fah*^*−/−*^ mice after NTBC withdrawal (Fig. 1d). These BMDHs were likely generated by fusion of donor WT BM-derived myelomonocytes with resident *Fah*^*−/−*^ hepatocytes, as they expressed both myelomonocyte markers (CD45, CD11b) and Fah (Fig. 1e and Supplementary Fig. S1). In addition, BMDHs exhibited a CD11b^+^Ly6C^–^Ly6G^–^F4/80^+^CD11c^–^ phenotype (Fig. 1f), identifying that liver-resident macrophages might be the major component within the myelomonocyte compartment to fuse with hepatocytes and allow their liver repopulation and survival.

**Figure 2:**
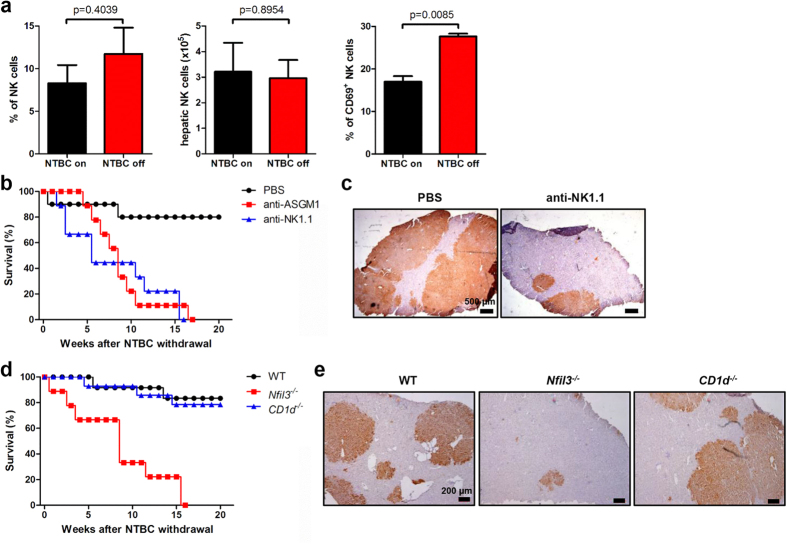
NK cells are essential for liver reconstitution. (**a**) Hepatic NK cell (CD3^−^NK1.1^+^) percentage, absolute number, and CD69 expression in *Fah*^*−/−*^ mice 16 weeks after WT BMT and 12 weeks after NTBC withdrawal (NTBC off); mice maintained on NTBC were used as controls (NTBC on). *Fah*^*−/−*^ mice transplanted with WT BM were treated with anti-ASGM1 mAb (*n* = 9), anti-NK1.1 mAb (PK136) (*n* = 9), or PBS control (*n* = 10) throughout the period of NTBC withdrawal. (**b**) Survival rate is shown. (**c**) Liver tissues were collected from mice in the PBS-or anti-NK1.1–treated groups 14 weeks after NTBC withdrawal, and immunohistochemical staining of Fah was performed (scale bar, 500 μm). (**d**) Survival rate of *Fah*^*−/−*^ mice transplanted with *Nfil3*^*−/−*^ (*n* = 9), *CD1d*^*−/−*^ (*n* = 14), or WT (*n* = 12) BM. (**e**) Immunohistochemical staining of Fah (scale bar, 200 μm) in liver tissues from *Fah*^*−/−*^ mice that received BMT from the indicated mouse strain 16 weeks after NTBC withdrawal. Representative data from 2 or 3 independent experiments are shown as the mean ± SEM.

To investigate the local immune response in the liver during BMDH generation, we evaluated hepatic lymphocyte composition and activation after BMT. NTBC withdrawal (NTBC-off condition), which promotes BMDH generation, resulted in dramatic upregulation of CD69 expression of hepatic NK cells (Fig. 2a and Supplementary Fig. S2a), but not T cells (Supplementary Fig. S3a, b), in the livers of BM-transplanted *Fah*^*−/−*^ mice compared to control mice continuously receiving NTBC (NTBC-on condition), even though the number and percentage of NK cells did not change (Fig. 2a and Supplementary Fig. S2a). This result indicated the possible involvement of NK cells during BMDH generation. We also found that NK cell depletion by anti-ASGM1 antibody treatment led to decreased survival and BMDH generation in WT BM-transplanted *Fah*^*−/−*^ mice, which was further confirmed by NK cell depletion with anti-NK1.1 antibody (Fig. 2b,c and Supplementary Fig. S4). Since anti-NK1.1 antibody also depleted NKT cells, we specifically tested whether NK or NKT played a role in this process by transplanting *Fah*^*−/−*^ mice with either *Nfil3*^*−/−*^ or *CD1d*^*−/−*^ BM, which led to NK or NKT deficiency^19^,^20^, respectively. Interestingly, *Nfil3*^*−/−*^, but not *CD1d*^*−/−*^, BM lost the ability to rescue *Fah*^*−/−*^ mice from liver failure since *Nfil3*^*−/−*^ BMT markedly decreased *Fah*^*−/−*^ mouse survival and Fah^+^ BMDH generation after NTBC withdrawal (Fig. 2d,e). This result suggested that NK, but not NKT, cells were essential for BMDH generation. We additionally determined that CD4^+^ or CD8^+^ T cells were also not required for BMDH generation, as expected (Supplementary Fig. S3c, d).

### NK cell–derived IFN-γ plays a critical role in hepatic reconstitution after BMT

**Figure 3:**
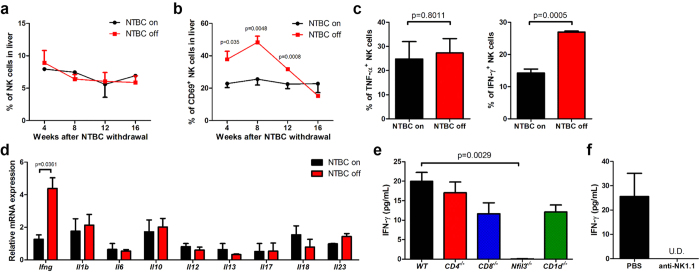
NK-derived IFN-γ increases after NTBC withdrawal. *Fah*^*−/−*^ mice were transplanted with WT BMCs, and hepatic NK cell percentage (**a**) and CD69 expression (**b**) were evaluated at the indicated time points by flow cytometry. (**c**)Intracellular cytokine expression in hepatic CD3^–^NK1.1^+^ NK cells was analyzed 8 weeks after NTBC withdrawal (NTBC off); mice maintained on NTBC were used as controls (NTBC on). (**d**) Cytokine mRNA expression in liver tissues of BM-transplanted *Fah*^*−/−*^ mice was measured by quantitative PCR 8 weeks after NTBC withdrawal. (**e**) *Fah*^*−/−*^ mice were transplanted with *CD4*^*−/−*^, *CD8*^*−/−*^, *Nfil3*^*−/−*^, or *CD1d*^*−/−*^ BMCs, and serum IFN-γ levels were evaluated by ELISA 12 weeks after NTBC withdrawal. (**f**) *Fah*^*−/−*^ mice transplanted with WT BM were treated with anti-NK1.1 mAb or PBS control throughout NTBC withdrawal, and serum IFN-γ levels were detected by ELISA 17 weeks after NTBC withdrawal. U.D., undetectable; Representative data from 2 or 3 independent experiments are shown as the mean ± SEM.

Since we observed that NK cells might be involved in BMDH generation from observing higher NK cell activation at a single time point (12 weeks) in Fig. 2a, we monitored NK cells over time by evaluating hepatic NK cell kinetics during BMDH generation. Although the percentage and total number of NK cells remained similar over time (Fig. 3a and Supplementary Fig. S2b), CD69 expression was greatly induced on NK cells and remained elevated for more than 3 months after NTBC withdrawal (Fig. 3b and Supplementary Fig. S2c). IFN-γ is an important effector molecule produced by NK cells as well as by NKT and conventional CD4^+^ and CD8^+^ T cells, among others. Consistent with the presence of more highly activated NK cells, intracellular IFN-γ in NK cells and liver tissue–derived *Ifng* mRNA were prominently increased after NTBC withdrawal (Fig. 3c,d and Supplementary Fig. S2d), while other measured cytokines did not change. Moreover, serum IFN-γ was detected in WT, *CD1d*^*−/−*^, *CD4*^*−/−*^, and *CD8*^*−/−*^ BM-reconstituted liver, but not in *Nfil3*^*−/−*^ BM-reconstituted liver (Fig. 3e), suggesting that the IFN-γ produced during this process was mainly derived from NK cells. Supporting this finding, NK1.1^+^ cell depletion abolished IFN-γ production after WT BM reconstitution (Fig. 3f). Collectively, these results suggest that NK cells are a major producer of IFN-γ in BM-reconstituted livers, prompting us to hypothesize that IFN-γ may play a role during liver regeneration by BMDHs after BMT.

**Figure 4:**
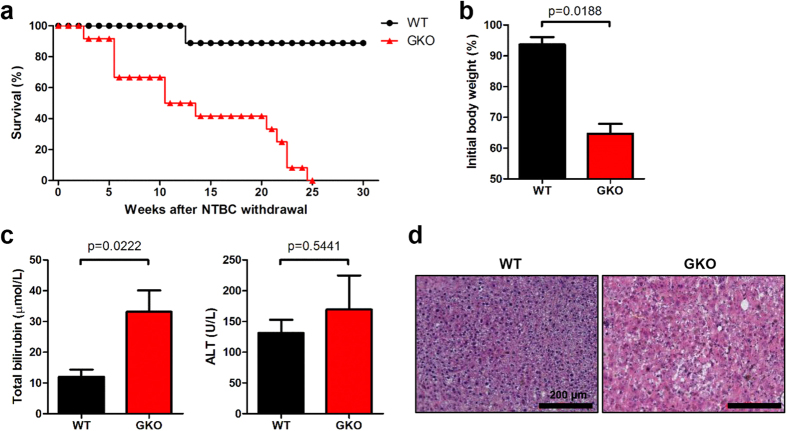
GKO BM cannot rescue *Fah*^*−/−*^ mice after NTBC withdrawal. *Fah*^*−/−*^ mice were transplanted with GKO (*n* = 12) or WT (*n* = 9) BMCs. (**a**)Survival rate was monitored, and (**b**)weight changes in GKO or WT BM-transplanted *Fah*^*−/−*^ mice were evaluated 22 weeks after NTBC withdrawal. (**c**) Total serum bilirubin and ALT levels were detected and (**d**) Liver tissues were collected for H&E staining 22 weeks after NTBC withdrawal (scale bar, 200 μm).

To address whether IFN-γ played an important role in BMDH generation after BMT, we transplanted IFN-γ–deficient (GKO) BM into *Fah*^*−/−*^ mice. Compared to WT BM-transplanted *Fah*^*−/−*^ mice that remained healthy after NTBC withdrawal and exhibited a high survival rate, GKO BM-transplanted *Fah*^*−/−*^ mice showed marked weight loss and died within 6 months after NTBC withdrawal (Fig. 4a,b); these mice also displayed severe liver dysfunction, with increased total serum bilirubin levels and liver pathology (Fig. 4c,d). Thus, GKO BMCs failed to rescue *Fah*^*−/−*^ mice, indicating that IFN-γ was required for BMDH generation after BMT.

**Figure 5:**
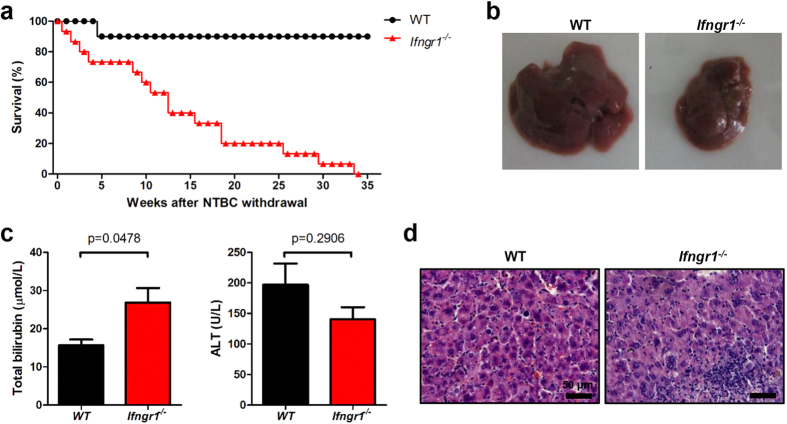
*Ifngr1*^*−/−*^ BM cannot restore liver function. *Fah*^*−/−*^ mice were transplanted with *Ifngr1*^*−/−*^ (*n* = 15) or WT (*n* = 10) BMCs, and (**a**) survival rate was monitored. At week 19 after NTBC withdrawal (NTBC off), (**b**) livers were grossly evaluated (representative pictures of whole livers are shown), and (**c**) total serum bilirubin and ALT levels were measured. (**d**) H&E staining from liver sections to evaluate liver pathology are shown (scale bar, 50 μm).

Next, we sought to identify the IFN-γ–responding cells in BM-mediated liver reconstitution. To first distinguish whether the IFN-γ–responding cells were the host hepatocyte or the BM-derived myelomonocytes, *Fah*^*−/−*^ mice were transplanted with *Ifngr1*^*−/−*^ BM so that the transplanted myeloid-derived cells rather than resident hepatocytes lost responsiveness to IFN-γ. Similar to transplantation with GKO BM, we observed that IFN-γR1–deficient BMT could not rescue survival of *Fah*^*−/−*^ mice (Fig. 5a). *Ifngr1*^*−/−*^ BM reconstitution also resulted in visible liver atrophy and necrosis (Fig. 5b), increased serum level of total serum bilirubin levels (Fig. 5c), and higher inflammatory cell infiltration into the liver (Fig. 5d) compared to WT BM reconstitution. This result suggested a “BM-intrinsic” requirement for IFN-γ signaling during BMDH generation.

### The IFN-γ–IFN-γR interaction contributes to the myelomonocyte–hepatocyte fusion process

**Figure 6:**
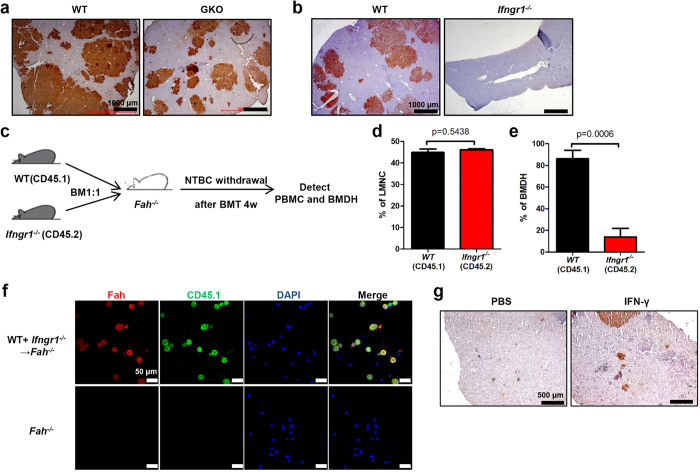
IFN-γ–IFN-γR interaction contributes to myelomonocyte–hepatocyte cellular fusion *in vivo*. (**a,b**) Liver tissues of *Fah*^*−/−*^ mice transplanted with GKO(**a**) or *Ifngr1*^*−/−*^ (**b**) BMCs were collected 20 weeks after NTBC withdrawal for immunohistochemical staining of Fah (scale bar, 1000 μm). (**c**) *Fah*^*−/−*^ mice(CD45.2) were transplanted with a 1:1 mixture of WT (CD45.1) and *Ifngr1*^*−/−*^ (CD45.2) BMCs. (**d,e**) The percentage of WT or *Ifngr1*^*−/−*^ cells within liver mononuclear cells (LMNC) (**d**) or CD11b^+^ hepatocytes(BMDH) (**e**) are shown 26 weeks after NTBC withdrawal. (**f**) Hepatocytes were separated from *Fah*^*−/−*^ mice 20 weeks after mixed BMT and stained for Fah (red) and CD45.1 (green). Nuclei were counterstained with DAPI (blue) (scale bar, 50 μm). (**g**) *Fah*^*−/−*^ mice transplanted with WT BM were treated with 500ng IFN-γ or PBS control for six weeks, liver tissues were collected 12 weeks after NTBC withdrawal, and immunohistochemical staining of Fah was performed (scale bar, 500 μm).

In line with the findings that IFN-γ or IFN-γR BMCs failed to rescue the *Fah*^*−/−*^ mice, Fah^+^ BMDH generation was dramatically reduced in those GKO or *Ifngr1*^*−/−*^ BM-reconstituted *Fah*^*−/−*^ mice compared to WT BM-reconstituted *Fah*^*−/−*^ mice (Fig. 6a,b). This reduction of BMDH formation was not likely due to the inhibition of BMDH proliferation, as PCNA staining showed no difference in the proliferation of Fah^+^ BMDHs between WT BM and GKO BM-reconstituted *Fah*^*−/−*^ mice (Supplementary Fig. S5a). The fusion of myelomonocytes with resident hepatocytes was previously reported to be the main mechanism for generating BMDHs^7^,^8^,^9^,^10^. We therefore wondered whether the IFN-γ–IFN-γR interaction played a role in this process. First, we determined that IFN-γ did not affect the generation or differentiation of the fusion partners-myelomonocytes from the BM, as no differences in percentage, number, or subsets of hepatic myelomonocytes were observed in GKO or *Ifngr1*^*−/−*^ BM-reconstituted *Fah*^*−/−*^ mice compared to WT BM-reconstituted *Fah*^*−/−*^ mice (Supplementary Fig. S5b–d). We then hypothesized that IFN-γ was required for stimulating the fusion between BM-derived myelomonocytes and resident hepatocytes. To address this hypothesis, we performed a mixed BM–reconstitution experiment in which BM cells containing a 1:1 mixture of CD45.1^+^ WT and CD45.2^+^ IFN-γR–deficient BMCs were transplanted into *Fah*^*−/−*^ mice (Fig. 6c). The majority of CD11b^+^ BMDHs in these mixed BM–chimeric *Fah*^*−/−*^ mice expressed the CD45.1 marker, indicating that they were derived from WT BM (Fig. 6d,e and Supplementary Fig. S6); importantly, this was likely due to the inability of IFN-γR–deficient myelomonocytes to fuse with the resident hepatocytes rather than an effect of IFN-γ on myelomonocyte survival or migration to the liver, as the similar percentage of CD45.1^+^ and CD45.2^+^ cells in the liver mononuclear cells (Fig. 6d) indicated equivalent reconstitution and hepatic migration of these cells after NTBC withdrawal. Immunofluorescence staining of BMDHs also showed that most of the Fah^+^ hepatocytes expressed CD45.1 (Fig. 6f), further suggesting that BM-intrinsic IFN-γR signaling was required for myelomonocytes to fuse with resident hepatocytes for BMDH generation. Finally, to investigate if IFN-γ could enhance the BMDH production *in vivo*, Fah^*−/−*^ mice transplanted with WT BMCs were treated with recombinant IFN-γ or PBS as control, a profound formation of Fah^+^ BMDH was observed in IFN-γ treated mice, whereas few BMDHs can be seen in controls at that time (Fig. 6g), suggesting that IFN-γ treatment could potentially accelerate the generation of BMDH during BMT-based therapy.

**Figure 7:**
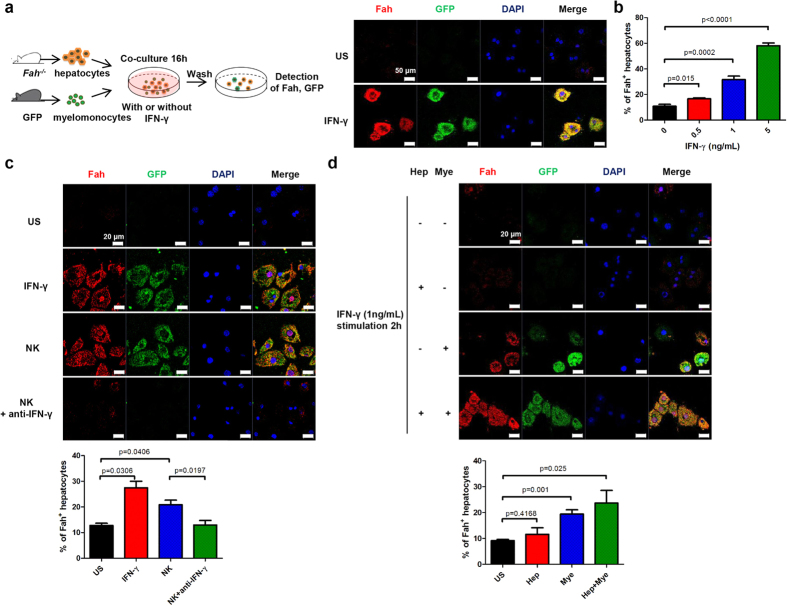
IFN-γ–IFN-γR interaction contributes to cellular fusion *in vitro*. (**a**) *Fah*^*−/−*^ hepatocytes were co-cultured with GFP^+^ splenic myelomonocytes for 16 h in the absence (unstimulated [US], top) or presence (bottom) of 1 ng/mL IFN-γ and then stained for Fah (red) and DAPI (blue) (scale bar, 50 μm). (**b**) Fah-positive hepatocytes were enumerated in the co-culture system from 5 random fields per section. (**c**) *Fah*^*−/−*^ hepatocytes were co-cultured with GFP^+^ splenic myelomonocytes for 16 h with medium alone (US), IFN-γ (1 ng/mL), NK cells pre-stimulated with 200 IU/mL IL-2 for 24 h, or pre-stimulated NK cells plus a neutralizing anti–IFN-γ Ab (20 μg/mL); cells were then stained for Fah (red) and DAPI (blue) (scale bar, 20 μm). Representative pictures and percentage of Fah^+^ hepatocytes from at least 2 independent experiments are shown. (**d**) *Fah*^*−/−*^ hepatocytes (Hep) or GFP^+^ splenic myelomonocytes (Mye) were pre-stimulated with (+) or without (−) IFN-γ (1 ng/mL) for 2 h and co-cultured together for additional 14 h. Representative images from at least 2 independent experiments for immunofluorescence staining of Fah (red) and GFP (green) and enumeration of Fah^+^ hepatocytes are shown(scale bar, 20 μm).

To confirm our *in vivo* findings regarding the essential role of IFN-γ in fusing myelomonocytes and hepatocytes together to generate BMDHs, we determined whether IFN-γ could also promote cell fusion *in vitro* by co-culturing GFP^+^ splenic myelomonocytes with *Fah*^*−/−*^ hepatocytes in the presence or absence of IFN-γ. Indeed, IFN-γ promoted fusion between myelomonocytes and *Fah*^*−/−*^ hepatocytes in a dose-dependent manner as evidenced by the emergence of GFP and Fah expression in *Fah*^*−/−*^ hepatocytes (Fig. 7a,b and Supplementary Fig. S7a). The cell fusion was further evidenced by the fact that co-culturing female *Fah*^*−/−*^ hepatocytes with male myelomonocytes in the presence of IFN-γ gave rise to Fah and Y chromosome positive cells (Supplementary Fig. S7b). In addition, these fusion derived Fah expressing cells were also positive for cytokeratin (CK)-18, a hepatocyte specific marker (Supplementary Fig. S7c), identifying they were hepatocytes rather than myelomonocytes. Moreover, we found that pre-activated NK cells also promoted cell fusion specifically through IFN-γ secretion (Fig. 7c). Also consistent with the findings from our *in vivo* experiments, we confirmed that IFN-γR signaling was required for myelomonocyte–hepatocyte cell fusion, as only IFN-γ–stimulated myelomonocytes, but not IFN-γ–stimulated hepatocytes, promoted the fusion of CD11b^+^ cells with *Fah*^*−/−*^ hepatocytes *in vitro* (Fig. 7d).

## Discussion

Clinical evidence suggests that BMT may provide a potential treatment for liver failure by regenerating liver structure and function through fusion of BM-derived myelomonocytes and liver-resident hepatocytes to generate BMDHs, which can then repopulate the liver; however, the mechanism underlying this fusion process is incompletely understood. The present study sheds light on this fusion process, as our data demonstrate that NK cell-derived IFN-γ can directly act on donor BM-derived myelomonocytes to promote fusion with host hepatocytes, thus facilitating BMDH generation during liver regeneration after BMT. To our knowledge, this is the first study to reveal the link between innate immunity and BMDH formation, and this information may be beneficial for understanding and improving the therapeutic intervention of BM-based therapy for liver failure.

Using a partial hepatectomy model, previous studies show that NK cells are involved in liver regeneration through immune-mediated hepatocyte repopulation^13^,^14^. Our results now show that NK cells can also promote BMDH formation in a BM-mediated model of liver regeneration. Although we did not observe any changes in the frequency of total NK cells during liver regeneration, it remained possible that a special subset of NK cells accumulated in the liver and secreted higher levels of IFN-γ. As NK cells were also derived from the BM in our model, it might be interesting to investigate whether the distinct hepatic environment created during liver reconstitution affected NK cell development. Hepatic NK cells remained highly activated for a long period after NTBC withdrawal, but the mechanism underlying how these NK cells became activated and maintained this activation needs further investigation. We speculate that dying hepatocytes may release many different types of self molecules into the extracellular environment, triggering inflammation by damage associated molecular patterns (DAMPs)^21^. In turn, liver-resident immune cells, such as macrophages, may recognize some of these DAMPs and subsequently activate NK cells by expressing ligands specific for NK cell–activating receptors or secreting NK cell–stimulating cytokines.

Our findings suggesting that NK cells promote liver repopulation in an IFN-γ–dependent manner impose a central role for IFN-γ during BMDH generation. According to previous reports as well as our own data presented here, we suppose that the BM-derived myelomonocyte cells in the liver that fused with hepatocytes were likely macrophages, which are already known to have the ability to fuse with other cell types. For example, the multinucleated giant cells found in tuberculosis granulomas originate from the fusion of monocyte- and macrophage-lineage cells^22^. Interestingly, IFN-γ is known to play an important role in promoting macrophage activation and is a prerequisite for macrophage fusion during the formation of multinucleated giant cells. IFN-γ can also initiate the fusion between monocytes and macrophages to develop Langhans giant cells, or between human alveolar macrophages and osteoclasts^23^,^24^,^25^. Here, we show that IFN-γ also participates in the macrophage–hepatocyte fusion process to generate BMDHs, although the molecular mechanism underlying how IFN-γ promotes fusion in our model remains unclear. Toward this end, further investigation into some IFN-γ–stimulating adhesion molecules that promote cell clustering and cell-to-cell adhesion/fusion, such as ICAM-1^23^, MFR, CD47, and CD44^26^, may yield some answers.

We examined in this study whether other IFN-γ–producing lymphoid cells, such as T and NKT cells, played a role in BMDH generation in our model. We found that T cells were not essential for BMDH generation, consistent with findings from a previous study showing that transplanted *Rag2*^*−/−*^ BM could still effectively generate BMDHs in *Fah*^*−/−*^ mice^10^. Additionally, mice transplanted with NKT-deficient *CD1d*^*−/−*^ BM retained the ability to reconstitute livers in *Fah*^*−/−*^ mice similar to WT BMT, indicating that at least CD1d-restricted NKT cells were also dispensable for BMDH generation. A previous study demonstrated that lymphoid cells did not participate as fusion partners to hepatocytes during BMDH generation by transplanting *Rag2*^*−/−*^ *γc*^*−/−*^ BM into *Fah*^*−/−*^ hosts^9^, which is consistent with our conclusions here. However, it also showed that the number of Fah^+^ nodules were similar between *Rag2*^*−/−*^ *γc*^*−/−*^ BM- and WT BM-transplanted mice. We noted that approximately 15% of the autologous BMCs remained in the irradiated recipient mice in this study, providing a potential source of IFN-γ from the host mice themselves. Additionally, it showed no difference between *Rag2*^*−/−*^ *γc*^*−/−*^ BM- and WT BM-transplanted mice from only a single time point 6 months after BMT; since no time kinetics for BMDH generation were provided, it remained possible that lymphocytes might have accelerated BMDH generation at an earlier time point than 6 months. In our present study, we tested a full complement of the kinetics underlying BMDH generation in the absence or presence of NK cells or IFN-γ, thus providing more comprehensive information to uncover the importance of extrinsic factors, such as innate cells or cytokines, in BMDH generation. Noteworthy, as there was no specific marker of donor derived cells for detecting the hematopoietic chimerism in our BMT experiments, the possibility that different bone marrow donors give rise to different degrees of hematopoietic chimerism cannot be excluded, which might directly affect the efficiency of cell fusion.

BMT has been used to treat patients with severe liver disease, but its efficacy is still limited by the low efficiency of BMDH generation^27^,^28^. Our findings that NK cells or IFN-γ can facilitate myelomonocyte–hepatocyte fusion and promote BMDH generation may be a great benefit to improving the therapeutic efficiency of BMT treatment. As several studies have shown that BMT can alleviate liver dysfunction and prolong survival of patients with end-stage liver diseases, improved myelomonocytes/hepatocyte fusion during this therapy could further decrease the apoptosis of hepatocytes and maintain liver function. Moreover, this type of therapy has great potential for treating liver diseases induced by genetic defects in hepatocytes, e.g. Wilson disease, hereditary fructose intolerance, α1-antitrypsin deficiency and hereditary tyrosinemia. Improved myelomonocytes/hepatocyte fusion could correct these genetic defects by “reprogramming” hepatocytes to cure these diseases. In addition, we could also image that improved myelomonocytes/hepatocyte fusion may be used as an alternative method for liver-directed gene transfer, this method should be better than engineering adenovirus vector based liver-directed gene therapy in safety. Taken together, our study provides not only new insights into the crosstalk between innate immunity and hepatocytes during BMDH generation and liver repair, but also new implications for BMT-based treatment of severe liver failure.

## Methods

### Mice and BM transplantation

*Fah*^*−/−*^ mice on 129S4 background kindly provided by Dr. Xin Wang (Inner Mongolia University, Hohhot, China) were used as the transplant recipients for all BMT experiments. *Fah*^*−/−*^ mice were continuously treated with NTBC in the drinking water at a concentration of 7.5 mg/L (a gift from Dr. Xin Wang). *Ifng*^*−/−*^ (GKO) and *Ifngr1*^*−/−*^ mice were obtained from the Model Animal Research Center (Nanjing, China). CD45.1^+^ mice were purchased from The Jackson Laboratory (Bar Harbor, ME, USA). *Nfil3*^*−/−*^ mice were kindly provided by Dr. Tak W. Mak (University of Toronto, Toronto, Ontario, Canada). *CD1d*^*−/−*^, *CD4*^*−/−*^, and *CD8*^*−/−*^ mice were kind gifts from Dr. Li Bai and Dr. Zhexiong Lian (University of Science and Technology of China, Hefei, China), all those donor mice were on B6 background. For BMT, male *Fah*^*−/−*^ mice (8–10 weeks old) were lethally irradiated with a total dose of 1000cGy, and 2 × 10^6^ BMCs in a 200 μl volume were intravenously injected 4 hours later. After adoptive cell transfer, *Fah*^*−/−*^ mice were maintained on NTBC for 4 weeks until NTBC was withdrawal to induce hepatocyte selection. All animals were housed in a specific pathogen-free facility and all the procedures regarding animal handling were performed in accordance with the animal care regulations of the University of Science and Technology of China. All the animal experiment protocols were approved by the Local Ethics Committee for Animal Care and Use at University of Science and Technology of China.

### Reagents and antibodies

The following antibodies were purchased from BD Biosciences (San Jose, CA, USA) and used for flow cytometry analysis: FITC-conjugated anti-Ly6C, anti-CD69, anti-CD11b, and anti–TNF-α; PE-conjugated anti-Ly6G, anti-CD8, and anti–IFN-γ; PerCP-Cy5.5–conjugated anti-CD3; PE-Cy7–conjugated anti-NK1.1 and anti-CD45; allophycocyanin(APC)-conjugated anti-CD45.2, and anti-CD11c; APC-Cy7–conjugated anti-CD4 and anti-CD45.1. APC-conjugated anti-F4/80 were purchased from eBioscience (San Diego, CA, USA). Stained cells were collected using a BD LSR II (BD Biosciences) flow cytometer, and data were analyzed using FlowJo 7.6 software (Tree Star, Ashland, OR, USA). For NK cell depletion, mice were injected with 30 μl anti-ASGM1 antibody once a week (Wako Co., Tokyo, Japan). For NK1.1^+^cell depletion, mice were intraperitoneally injected with 200 μg anti-NK1.1 (Clone PK136, purified from ascites).

### Liver function tests

Serum was assayed for total bilirubin and alanine aminotransferase (ALT) levels using commercially available diagnostic kits (Rong Sheng, Shanghai, China) with a Chemray 240 (Rayto, Shenzhen, China) automated chemistry analyzer.

### Histological and immunohistochemical analysis

Liver samples were fixed in 10% phosphate-buffered formalin and embedded in paraffin wax. Tissue sections (5-μm thick) were rehydrated and stained with hematoxylin and eosin. For immunohistochemical analysis, sections were incubated in 3% H_2_O_2_ for peroxidase blocking and in goat serum for serum blocking. Liver sections were then stained with a polyclonal rabbit antibody to mouse Fah (Abnova, Taipei City, Taiwan) in a 1:5000 dilution overnight at 4 °C. The biotinylated goat anti-rabbit IgG secondary antibody was then added, followed by avidin-coupled peroxidase (ZSGB-BIO, Beijing, China). Color was developed with the commercially available DAB kit according to the manufacturer’s instructions (Vector Laboratories, Burlingame, CA, USA). For fluorescent staining, Cy3-conjugated goat anti-rabbit IgG (Abcam, Cambridge, MA, USA) at a 1:200 dilution was used as the secondary antibody. Nuclear DNA was stained with 1 μg/mL DAPI (Santa Cruz Biotechnology, Santa Cruz, CA, USA).

### Cytogenetics

Y chromosomes were detected by fluorescence *in situ* hybridization using SpectrumGreen dUTP labeled mouse Y chromosome paints (clone pY353/B) as described before^7^.

### ELISA for Cytokine Detection

Serum IFN-γ concentration was determined by mouse IFN-γ enzyme-linked immunosorbent assay kit (DAKEWE, Beijing, China) according to the manufacturer’s instructions.

### Isolation of hepatocytes

Briefly, mice were anesthetized, the livers were perfused with 0.5 mM EGTA solution through portal vein and followed by *in situ* digestion with 0.075% collagenase I solution (Sigma-Aldrich). Tissue debris was removed through repeated spinning at 50 g for 1 min, the hepatocytes were then pelleted after a density gradient centrifugation on 40% Percoll solution for 10 mins at 400 g.

### Isolation of Liver Mononuclear Cells

Liver Mononuclear Cells (MNCs) were isolated by passing the livers through a 200-gauge stainless steel mesh. The cells were pelleted, resuspended in 40% Percoll, and gently overlaid onto a 70% Percoll gradient. After centrifuging at 1,260 g for 30 minutes, liver MNCs were collected from the interphase.

### Co-culture system

*Fah*^*−/−*^ hepatocytes (1 × 10^5^) were placed onto 24-well plates precoated with collagen. Four hours after plating, cell culture supernatant was removed, leaving only adherent hepatocytes in the well. Splenic CD11b^+^ cells were then added to the wells and co-cultured with the adherent hepatocytes for 16 hours at a 1:1 ratio; in some cases, recombinant mouse IFN-γ (Peprotech, Rocky Hill, NJ) or 5 × 10^4^ NK cells were added to the co-culture. For neutralizing IFN-γ, 20 μg/ml anti–IFN-γ Ab (Biolegend, CA, USA) were adding to the culture system.

### Quantitative PCR

Total RNA was isolated from liver tissue and reverse transcribed into cDNA. Quantitative PCR was measured by the SYBR Green (TaKaRa, Dalian, China) staining method according to the manufacturer’s instructions. Gene expression levels were calculated relative to the housekeeping gene *Gapdh*. The PCR primer sequences used in this study are as follows: *Gapdh*, forward 5′-CAATGTGTCCGTCGTGGA-3′ and reverse 5′-GATGCCTGCTTCACCACC-3′; *Ifng*, forward 5′-CTCTGAGACAATGAACGCTACA-3′ and reverse 5′-TCTTCCACATCTATGCCACTT-3′; *Il1b*, forward 5′-CTCCATGAGCTTTGTACAAGG-3′ and reverse 5′-TGCTGATGTACCAGTTGGGG-3′; *Il6*, forward 5′-TCCAGTTGCCTTCTTGGGAC-3′ and reverse 5′-GTGTAATTAAGCCTCCGACTTG-3′; *Il10*, forward 5′-ATGCCTGGCTCAGAC-3′ and reverse 5′-GTCCTGCATTAAGGAGTCG-3′; *Il12*, forward 5′-CTCTGAGACAATGAACGCTACA-3′ and reverse 5′-CTCAGATAGCCCATCAC-3′; *Il13*, forward 5′-AGACCAGACTCCCCTGTGCA-3′ and reverse 5′-TGGGTCCTGTAGATGGCATTG-3′; *Il17*, forward 5′-TCAGACTACCTCAACCGTTCC-3′ and reverse 5′-GGTGGTCCAGCTTTCCCT-3′; *Il18*, forward 5′-ACTGTACAACCGCAGTAATAC-3′ and reverse 5′-AGTGAACATTACAGATTTATCCC-3′; *Il23*, forward 5′-AGCGGGACATATGAATCTACTAAGAGA-3′and reverse 5′-GTCCTAGTAGGGAGGTGTGAAGTTG-3′.

### Statistical analysis

Results were analyzed using a non-paired Student’s *t* test. Differences achieving *P* < 0.05 were considered to be statistically significant.

## Additional Information

**How to cite this article**: Li, L. *et al.* Natural Killer Cells-Produced IFN-γ Improves Bone Marrow-Derived Hepatocytes Regeneration in Murine Liver Failure Model. *Sci. Rep.* **5**, 13687; doi: 10.1038/srep13687 (2015).

## Supplementary Material

Supplementary Information
