# Supplementary material for: Natural Killer Cells-Produced IFN-γ Improves Bone Marrow-Derived Hepatocytes Regeneration in Murine Liver Failure Model
Source: Sci Rep. 2015 Sep 8;5:13687. doi: 10.1038/srep13687 (PMC4561890; doi:10.1038/srep13687)
Supplement: Supplementary Information [file srep13687-s1.pdf]

Natural Killer Cells-Produced IFN-γ Improves Bone Marrow-Derived Hepatocytes Regeneration in Murine Liver Failure Model

Authors: Lu Li[1], Zhutian Zeng[1], Ziping Qi[1], Xin Wang[3], Xiang Gao[4], Haiming Wei[1], Rui Sun[1,2], and Zhigang Tian[1,2*]

Supplementary Figures and legends:

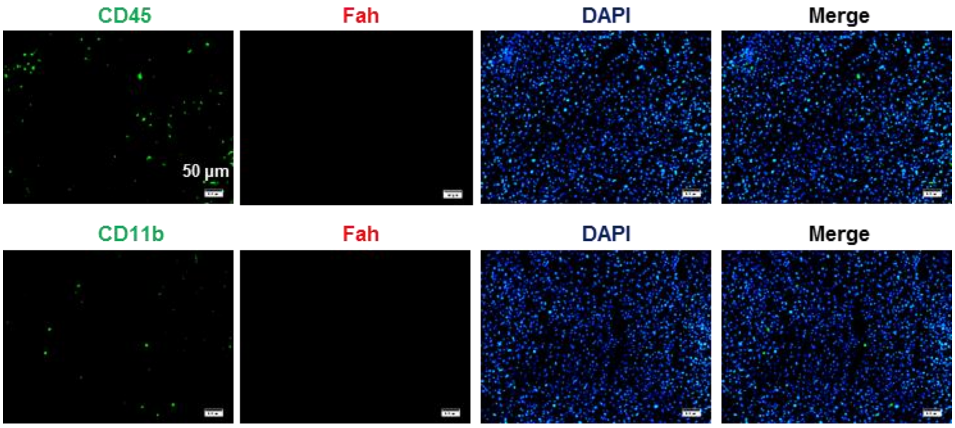

Supplementary Fig. S1. Immunofluorescence staining of liver tissues from control *Fah*$^{-/-}$ mice.

Liver tissues of *Fah*$^{-/-}$ control mice were collected and stained for Fah (red) and CD45 or CD11b (green) by immunofluorescence (scale bar, 50 μm).

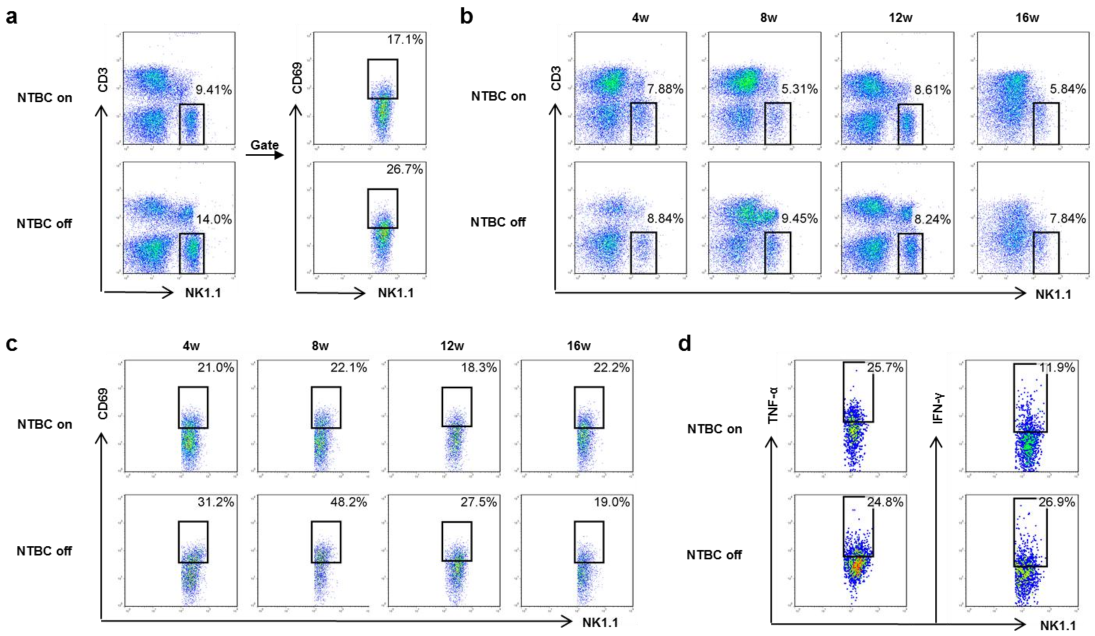

Supplementary Fig. S2. Representative FACS plots of NK cells frequencies and their activation states after NTBC withdrawal. (**a**) Representative FACS plots of Fig. 2a. (**b**) Representative FACS plots of Fig. 3a. (**c**) Representative FACS plots of Fig. 3b. (**d**) Representative FACS plots of Fig. 3c.

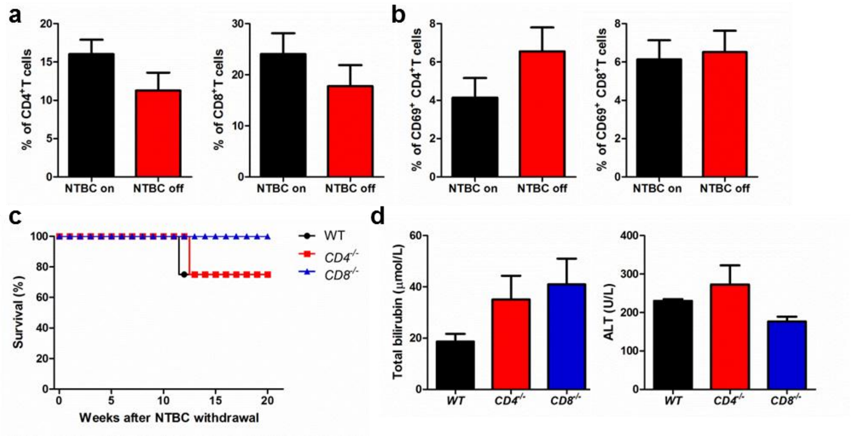

Supplementary Fig. S3. T cells are not involved hepatic reconstitution. Hepatic CD4^{+} and CD8^{+}T cell percentage (**a**) and CD69 expression (**b**) in *Fah*$^{-/-}$mice 16 weeks after WT BMT and 12 weeks after NTBC withdrawal. *Fah*$^{-/-}$mice were transplanted with *CD4*$^{-/-}$ (*n*=4), *CD8*$^{-/-}$ (*n*=4), or WT (*n*=4) BMCs, and (**c**) survival rate was monitored. (**d**) Total serum bilirubin and ALT levels were detected 16 weeks after NTBC withdrawal.

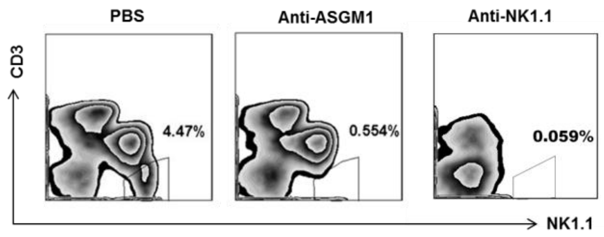

Supplementary Fig. S4. Validation of antibody mediated depletion of NK cells.

WT mice were treated with anti-ASGM1 or anti-NK1.1, hepatic NK cells ($CD3^{-}NK1.1^{+}$) were evaluated by flow cytometry 3 days later.

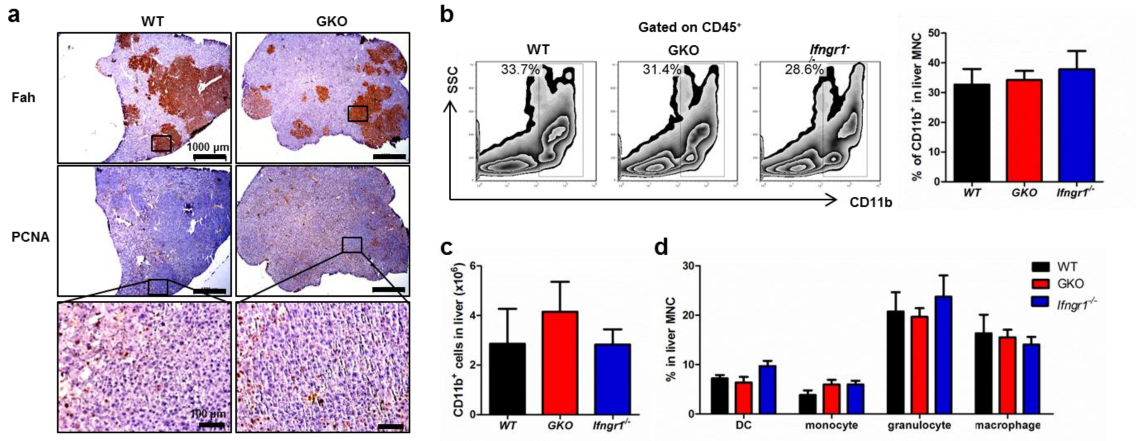

Supplementary Fig. S5. IFN-γ signaling deficiency does not alter expansion of BMDHs or liver myelomonocytic cell subsets. (**a**) Liver tissues of *Fah*$^{-/-}$ mice transplanted with GKO or WT BMCs were collected 20 weeks after NTBC withdrawal for immunohistochemical staining of Fah and PCNA (scale bar, 1000 μm for top and middle; 100 μm for bottom). (**b**) Four weeks after being transplanted with GKO or *Ifngr1*$^{-/-}$BMCs, hepatic mononuclear cells (MNCs) were isolated from *Fah*$^{-/-}$mice and examined by flow cytometry. The percentage of CD11b^{+} cells is shown. (**c**) Total CD11b^{+} cell number in liver MNCs. (**d**) The percentage of CD11c^{+} DCs, CD11b^{+}Ly6C^{+}Ly6G^{-} monocytes, CD11b^{+}Ly6C^{+}Ly6G^{+} granulocytes, and CD11b^{+}F4/80^{+} macrophages in liver MNCs. Data are expressed as the mean ±SEM.

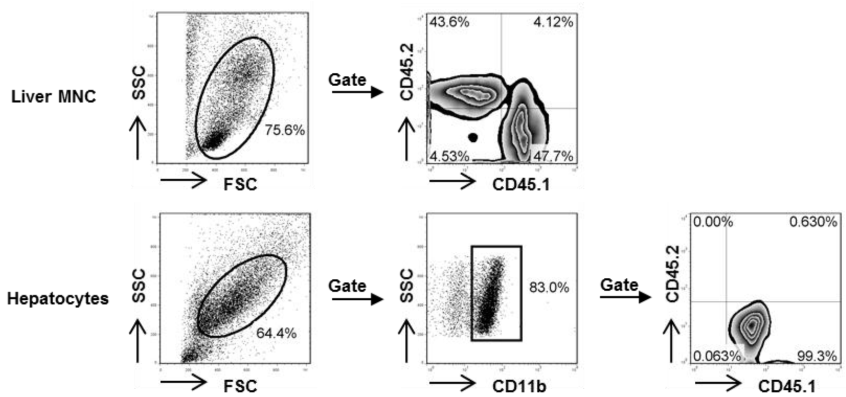

Supplementary Fig. S6. Gating strategy for Fig.6d-e

Liver Mononuclear Cells (MNCs) or hepatocytes of *Fah*$^{-/-}$mice transplanted with a 1:1 mixture of WT (CD45.1) and *Ifngr1*$^{-/-}$(CD45.2) BMCs were evaluated by flow cytometry.

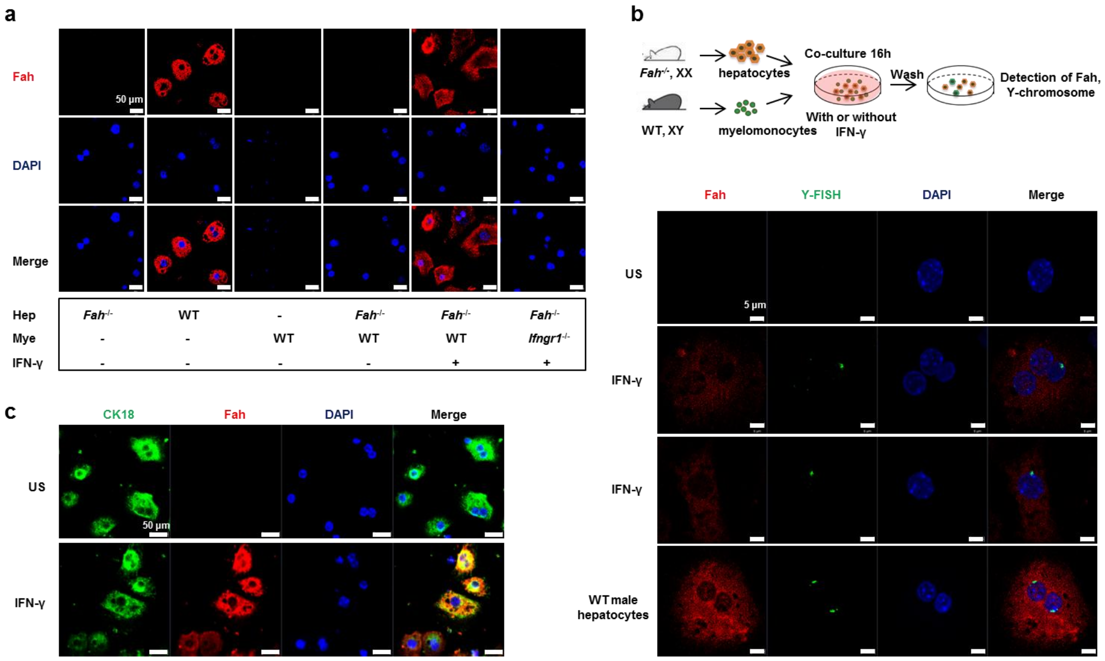

Supplementary Fig. S7. IFN-γ–IFN-γR interaction contributes to cellular fusion in vitro. (**a**) The controls of in vitro co-culture system were shown for Fah (red) and DAPI (blue) (scale bar, 50 μm). (**b**) Female *Fah*$^{-/-}$ hepatocytes were co-cultured with male splenic myelomonocytes for 16 h in the absence (unstimulated [US]) or presence of 1 ng/mL IFN-γ and then stained for Fah (red) and DAPI (blue), Y-chromosomes (green) were detected later by fluorescence in situ hybridization (scale bar, 5 μm). WT male hepatocytes were used as positive control. (**c**)*Fah*$^{-/-}$ hepatocytes were co-cultured with splenic myelomonocytes for 16 h in the absence (unstimulated [US], top) or presence (bottom) of 1 ng/mL IFN-γ and then stained for CK18 (green), Fah (red) and DAPI (blue) (scale bar, 50 μm).
